# Lifeboat ethics, risk, and therapeutic opportunity: an appeal for equitable psychedelic therapy access in the “high-risk” addiction patient

**DOI:** 10.3389/fpsyt.2023.1159843

**Published:** 2023-09-20

**Authors:** Taylor Black

**Affiliations:** Department of Psychiatry and Behavioral Sciences, University of Washington, Seattle, WA, United States

**Keywords:** psychedelic, policy, risk, equity, addiction, cost, psychiatric, ethics

## Abstract

Psychedelic-assisted treatment (PAT) for mental health is in renaissance. Psilocybin and MDMA stand near FDA approval, and US cities and states are decriminalizing or regulating the non-clinical use of psilocybin. However, neither FDA indications nor a regulated use model sufficiently address the complex needs and opportunities for an improved treatment of addiction. When paired with disability and social dispossession, addiction increasingly burdens informal care networks, public safety, and particularly healthcare systems. Stigma and mistreatment alienate people from opportunities for care and multiply the costs of providing care. This dynamic worsens socially determined resource limitations, enforcing stark ethical choices and perpetuating socioeconomic inequities, isolation, mental illness, medical illness, overdose, suicide, and violence. In order for psychedelic treatments to achieve their greatest utility to population health, we must intentionally develop regulatory, clinical, and payment systems supporting clinical research, rigorous safety monitoring, and implementation to address these immense needs and reduce the barriers to engagement for those who now bear the costs, including those who work at the front lines of addiction care. To achieve full fruition, I advocate for a collaborative approach, built from within networks of mutual social support but linked and accountable to public institutions charged with the equitable dissemination of these therapies for the greatest social and health equities. Rather than relegating PAT to the needs of the commercially insured or wellness markets, this is the moment to learn from ancient traditions of ritualized sacramental use, organized around faith in our mutual dependency and accountability, and to capture an opportunity to improve population health and equity. To miss this opportunity is to accept the status quo in the midst of a growing emergency, for lack of moral vision and intention to change our habits.

## Introduction

“Love? What is it? Most natural painkiller what there is.” *William S. Burroughs*

Addiction reflects and sustains social and health inequities and excludes the most ill from parity in care at an immense cost. A “social disease” model interrogates mechanisms (1) of social and developmental transmission via parental addiction (2), dislocation (3), and stigma (4). Adverse childhood experiences (ACEs) (5) drive somatic symptoms (6), mental illness (7), addiction (8), and treatment dropout (9). The socialized cost of addiction such as medical expenses (10, 11), early mortality (12), and criminality (13) is a total of nearly $1T annually in the USA (14). Despite increasing public spending (15) and high aspirations, the prevalence of addiction has doubled in one generation, now leading the world (16), and is likely to end in death rather than recovery (17).

“Safety-net” healthcare is a major venue of such alienation, despair, and self-destruction. A “revolving door” of incarceration (18), hospitalization (19), and distrustful healthcare experiences burns out patients and workers (20, 21). Forced to manage inequality, triaging high-risk and ethically complex situations, we rule a biomedical lifeboat of costly inefficiency while blaming the drowning for the tides. Our biomedical infra structure structurally fails (22) without social systems that promote belonging and recovery (23) for those with addiction.

Such systems demand mutual faith and intention. Traditional, legally-sanctioned “sincere” (24) religious groups provide safety (25–27) and regulate access (28) to sacramental psychedelic use, illustrating practical rituals of group continuity, mutual aid, intention, and preparation (29). Paired with clinical research, they should inform current policy questions around the implementation of equitable access to PAT. These cultural and legal histories are heterogenous, including psilocybin (30, 31), peyote (32–34), and ayahuasca (35), evolving in post-colonial communities sometimes in response to social disease and oppression (36) and the prevalent demands for mental health and addiction care (37–39). As the legality of PAT evolves, we should protect as such “sincere” new groups that adhere to the faith in human interdependency, cemented by the sacrament of intentional group psychedelic use.

## Case example

Mr. L's development is marked by the grit of poverty, neighborhood violence, and childhood sexual abuse by his stepfather, unacknowledged by his mother. He achieved an education, career, marriage, and parenthood, but attachment insecurity and nightmares pushed him toward sedative addiction and decades of isolation, incarceration, illness, indignity, and dependency.

I met him at about 60 years of age, a self-described “gutter-drunk” and emergency department high-utilizer, after a series of dangerous intoxications and withdrawal seizures when using “street Xanax” of inconsistent purity. His goal was to manage his chronic anxiety, quit using alcohol and illicit benzodiazepines, and rebuild family relationships. He had already burned through counselors, peers, case managers, short-term detoxes, and residential treatments focused on sobriety. “I want treatment that works.” We agreed to harm-reduction benzodiazepine maintenance (40) using clonazepam, buprenorphine, gabapentin, paroxetine, hydroxyzine, and prazosin, meeting regularly over two years of relative stability, warmth, and relationship growth among sober peers and with his children.

As studies proliferated, it was often wondered when or if we might access PAT to consolidate different schemas of vulnerability and self-worth that informed our many slips and boundary tests. Mr. L was trusting, motivated, wise, and tough. Then, the pandemic started to tear apart our fragile raft. Unable to quit smoking to get the hip arthroplasty and mobility he needed to cope adaptively to pain and isolation, these problems compounded into relapse after his stepfather's funeral. He suffered repeated brain injuries, over multiple nocturnal ED visits, quickly sobering and leaving to avoid our gaze. His children pulled away again, deepening his despair. His disorderly intoxication cost him eviction from a clean and sunny housing unit. At my last contact, he was cognitively impaired, barely surviving in public housing, hopelessly depressed, and using heavily.

Addiction impoverishes us all: Mr. L loses his identity, privacy, dignity, independence, and health; his clinical team is demoralized and traumatized; society bears the high cost of hospital visits and early-onset custodial care. Deprived of cure or comfort, I console myself by caring and witnessing. Such self-destruction is only meaningful, however, through intentional action to avert the next tragedy.

## Clinical and policy priorities

PAT for addiction remains experimental and, despite reassuring safety signals in non-clinical use (41, 42), may have greater risks. Intentional policy work now can provide for safe and equitable access as treatment data emerge. In addition to the 20th-century trials of LSD in alcohol use disorder (AUD) (43), there are new trial data proving significant benefits of psilocybin in AUD (44), smoking cessation (45), and two US trials in recruitment for stimulant use disorders (46). Ongoing phase—three studies for PTSD and MDD will conclude within the year, with anticipated FDA approval of MDMA and psilocybin (47) for clinical use by 2024. The cost-effectiveness analysis of MDMA for PTSD costs $12K per person for a favorable (48) $25K (49) per QALY. Typically excluded “high-risk” patients could also yield proportionally greater margins of benefit if the efficacy is comparable to low-risk patients.

Community-based participatory research (CBPR) clinics and coordinated registry networks (CRNs) (50) are necessary for the next quality improvement and implementation in PAT for addiction. They should have five functions:

1. Measure transparently and systematically in harmony with federal guidance for “harm reduction, risk mitigation, and safety monitoring” (51) to identify markers of differential risk and benefit to deftly maximize safety, economic (52), and public health benefits (53).
2. Provide access to higher-risk individuals in need of more complex care.
3. Innovate and implement cost-effective and accessible options.
4. Recruit and train diverse and highly effective psychedelic clinicians.
5. Collaborate with mutual-support groups to provide recovery-oriented PAT for step-down continuity care, where interpersonal accountability and peer support can replace institutional care and surveillance.

Various jurisdictions have decriminalized or regulated psychedelics for adult use (54). Regulated cannabis use illustrates the harms and opportunity costs of misdirected policies that promoted charlatanism (55), consumption-based businesses (56), health and psychosis risks concentrated in young socially dispossessed BIPOC men (57), and increased suicide and alcohol deaths (58). Whether concurrent declines in racially biased policing and incarceration (59) balance such harms may be “unanswerable” (60). The capitalized industry is patenting color schemes and soundtracks for private franchise PAT (61) serving the wellness “spa” market (62), while unregulated freelance “trip guides” or retreats of dubious accountability and safety (63, 64) may prioritize profit over beneficence. Lightly regulated or PAT might help many and harm few, but this commercialization severs the source of transformational, sacramental cultural power.

## Conclusion

People suffering the deep spiritual wounds of chronic and complex addiction are often both pariahs *and* exemplars of grit, resilience, wisdom, and grace and may benefit from PAT if they can be prepared for its rigors and supported through catharsis and integration of change. Self-sustaining peer groups modeled on sacramental psychedelic use offer affordable and accessible continuity and social accountability when embedded in CBPR or CRNs to guide the implementation of PAT, rigorously monitor individual outcomes, and maximize community health. Such bonds are the true medicine and always have been.

## Data availability statement

The original contributions presented in the study are included in the article/supplementary material, further inquiries can be directed to the corresponding author.

## Ethics statement

Written informed consent was obtained from the individual(s) for the publication of any potentially identifiable images or data included in this article.

## Author contributions

The author confirms being the sole contributor of this work and has approved it for publication.
